# Implementation intentions speed up young adults’ responses to prospective memory targets in everyday life

**DOI:** 10.1371/journal.pone.0260856

**Published:** 2022-01-18

**Authors:** Kaja Szarras-Kudzia, Agnieszka Niedźwieńska

**Affiliations:** 1 Department of Psychology, Jesuit University Ignatianum, Kraków, Poland; 2 Applied Memory Research Laboratory, Department of Psychology, Jagiellonian University, Kraków, Poland; Julius-Maximilians-Universität Würzburg, GERMANY

## Abstract

Prospective memory (PM), which is the ability to remember to do something in the future, is vitally important for successful everyday functioning. Although young adults demonstrate high PM abilities in laboratory settings, their abilities to complete intended actions in naturalistic settings are surprisingly low. The present study tested the effectiveness of various encoding techniques in improving young adults’ performance in everyday life. Ninety-two participants were asked to remember to take photographs of receipts for a duration of seven days. The task instructions were either given alone or followed by: (a) the if-then statement, (b) visualising the task, or (c) the combination of the if-then statement plus visualisation. The if-then statement alone significantly speeded up responses to the prospective memory targets, i.e., less time elapsed between getting a receipt and taking a photograph of it. With no effect of the if-then statement on the proportion of correct PM responses, the results may suggest that the if-then statement strengthened the PM cue-intention association but did not influence the PM cue saliency.

## Introduction

Prospective memory (PM) is the ability to remember to do something in the future, for example, posting a letter when seeing the post office (event-based PM) or taking a pill at 7:00 p.m. (time-based PM). A particular challenge in relation to succeeding in PM tasks is that the retrieval of the intended action (posting a letter) has to be self-initiated upon encountering a target event (seeing the post office), when one is usually engaged in other ongoing activities (going to work and thinking about job-related problems) [1]. Time-based PM tasks pose an even bigger challenge as the retrieval of the intended action cannot be facilitated by a target event, and effortful time-monitoring is required. It is because of the high cognitive demands of PM tasks and the fact that they constitute a significant part of an everyday routine, that remembering to carry them out is a daily struggle. In fact, more than sixty percent of memory failures that people report in diaries involve the forgetting of planned actions, and PM failures are significantly more frequent than forgetting information from the past and absent-minded failures [2,3]. Research on everyday PM failures show that they have a whole range of negative consequences, from wasted time and money, interpersonal troubles, to losses in the social perception of an individual’s reliability in both private and work-related contexts [3]. Another category of negative consequences of PM failures are those posing threats to health or life at the workplace, e.g., aviation [4,5] or healthcare workers [6,7].

One of the encoding techniques that may help people act effectively on their intentions is implementation intention (II). II integrates situational cues and the intended action into a specific sentence: ‘If situation X occurs, then I will perform Y’ [8–10]. The technique has proved to be helpful in the goal pursuit studies, in which it involved specifying exactly when and how the goal would be achieved (e.g., *when I am back from church on a Christmas day*, *then I will sit at my father’s desk and start writing a report*), in contrast to having just a goal intention (e.g., *I intend to write a report over Christmas holiday*) (e.g., [11–13]). II has been found to facilitate the initiation of goal-directed actions [11] and help when those actions require considerable effort, delaying gratification or cause discomfort, e.g., ensuring that you engage in a 20-minute session of intense exercise once a week [14,15].

In contrast to the tasks used in the goal-pursuit studies, PM tasks are already very well specified and the intended actions are neither difficult nor uncomfortable (e.g., *posting a letter* when seeing the post office, *validating the travel card* when getting to the train station). The PM tasks pose only a cognitive and mnemonic challenge, i.e., how not to forget to respond to a prespecified cue or situation when being engaged in other ongoing activities at that time. Despite these task differences, the benefits of using II for improving performance on PM tasks have been documented, especially for populations with poorer cognitive functioning, i.e., clinical groups with PM deficits (e.g., [16–20]) and older adults (e.g., [21–25]). For those populations, the benefits from II that have been found in laboratory-based studies seem to generalise to PM tasks performed in everyday life, at least for time-based tasks [22,26,27].

Much less work has been done to examine whether II affects PM in young adults even though, when it comes to the tasks performed in everyday life, it is young adults rather than middle-aged and older adults who struggle the most to remember to act on their intentions (e.g., [2,3,28,29]). The empirical data as to whether II may be effective in improving PM performance among young adults comes exclusively from laboratory-based studies, with most studies demonstrating the benefits of using II (e.g., [30–34]) and some studies that did not find any benefits ([35], Experiment 3, [36,37]). Particularly informative are those studies which, despite the common practice of combing the ‘if-then’ statement with visualisation, showed the effectiveness of the ‘if-then’ statement as an isolated strategy [24,38,39], compared with the standard PM instruction. The standard instruction usually had the form of a request to perform an additional PM task during the execution of the ongoing task. For example, when completing a multiple-choice test, participants may be asked: *If you see questions pertaining to ‘states*,*’ you should press the ‘6’ key* [24]. Two studies in which the standard PM instructions were compared with using either the if-then statement alone, visualisation alone, or the combination of the if-then statement with visualisation showed that all manipulations that were additional to the standard instructions improved PM performance to a similar extent ([24,39]; see [30] for a similar result). However, to our best knowledge, no studies so far have investigated whether the ‘if-then’ statement and other encoding techniques work equally well for PM tasks that young adults need to perform in everyday life.

It has been suggested [9,38,40] that, for PM tasks, II stimulates a strong associative encoding between an anticipated situation and an intended action, thereby promoting more reflexive and automatic triggering of the intended action when the critical situation is encountered. This proposal has been tested based on the assumption that if II reduces reliance on the controlled, resource-demanding processes and fosters relatively automatic retrieval, compared to the standard PM instruction, then performing a PM task will require less resources under the II instruction than under the standard PM instruction. The results of the studies that addressed this proposal are not unequivocal. When Cohen and Gollwitzer [40] compared response times for performing the ongoing activity alone and for performing the ongoing activity in the presence of a PM task, they found that, with II, the PM task did not exact a cost from the ongoing activity, but the costs were evident under the standard PM instruction. Similarly, McFarland and Glisky [24] found that the performance of the ongoing task was better when II was formed, compared to when the standard PM instruction was provided. However, when the resource availability for a PM task was directly limited by increasing the attentional demands of the ongoing activity, II prevented PM decline in some experiments [38], but not in others [35].

### The present study

The aim of the present study was to investigate, for the first time, whether the if-then statement and visualisation work for the PM performance of young adults in everyday life. First, we wanted to examine potential benefits of these encoding strategies in terms of whether young adults would more often remember to perform the intended action, and thereby the number of their accurate PM responses would increase. In contrast to most II studies in which the if-then statement was combined with other techniques, most often visualisation, we investigated the effects of the if-then statement alone and visualisation alone, as well as the effects of the if-then statement and visualisation combined. Second, we wanted to examine potential benefits of these strategies in terms of whether young adults would respond more quickly to a target event. The second aim follows the theoretical proposal that II promotes more automatic triggering of the intended action when the critical situation is encountered. PM research so far has measured the level of automaticity under the II instruction as a degree to which cognitive resources are consumed, with the assumption that more automatic processes require less resources. On the basis of differences between controlled and automatic processes (see [41] for a review), which specify automatic processing as being more fluent, more efficient, and, importantly, faster, we used response speed as a measure of automaticity. By doing so, we followed several PM studies in which both the number of correct responses and response speed were used as measures of PM performance [e.g., 42–46], as well as several II studies, outside PM domain, in which response speed was used to show that II leads to more automatic responses [e.g., 47–49].

When developing the experimental task, we were inspired by frequent everyday tasks in which success critically depends on time, i.e., when even short delays in responding to a prespecified cue or situation result in failure. For instance, the person needs to remember to validate the ticket as soon as they get on the bus or validate the travel card as soon as they get to the train station. Since immediate responding is required on these tasks (even a few minutes of delay may result in fines), they may pose a challenge even for young adults whose cognitive processes are at their peak. In contrast to all naturalistic studies that investigated the effects of II among older adults [22,26,27], and vast majority of studies on everyday PM in general, we wanted to use an event-based PM task. The common practice of using time-based tasks in naturalistic PM studies is due to the fact that, compared to event-based tasks, the objective measurement of everyday performance on time-based tasks is relatively easy. It is enough, for example, to check whether (and what time) the participant sent a text message that they had been asked to send to the experimenter at a specified time. For event-based tasks, the experimenter needs to know whether (and when) the target event, to which the participant had been asked to respond, appeared during the participant’s everyday routine.

Therefore, we faced the challenge of developing an event-based PM task that would enable a precise and objective measurement of not only whether the intended action was performed, but also how much time exactly elapsed between an occurrence of a target event and performing the intended action. To meet these demands, we developed a new naturalistic event-based task that required an immediate PM response under very precisely defined circumstances in everyday life. Specifically, we asked participants to remember to use their mobile phones to take a picture of the first receipt they would be given when shopping on a given day, for a duration of seven days. The task is based on the fact that receipts always include information about the exact time when they were supplied, whereas mobile phones have the capability to record the exact time when the photograph was being taken. Comparing those two times provides an exact measure of the response time in the PM task.

Based on the results of laboratory-based studies that examined the benefits from various encoding strategies for PM improvement in young adults, we expected that when the standard PM task instruction would be followed by any of the three encoding strategies (the if-then statement, the visualisation of carrying out the PM task, and the combination of the two) PM performance would improve, i.e. participants would more often remember to make a photograph of a receipt. As laboratory-based studies rather consistently show that those three encoding strategies improve PM performance to a similar extent [24,30,39], we did not formulate hypotheses regarding the differences in PM performance between the conditions in which different types of encoding strategies were used. Based on the results of the laboratory-based studies showing that PM tasks completed under the II instruction are less resource-consuming [24,38,40], as well as the empirical data showing that automatic processes are faster than controlled processes (e.g., [50–52]), we also expected that accurate PM responses would appear faster when the standard PM instruction would be followed by any of the three encoding strategies, i.e., less time would elapse between an occurrence of the target event (getting a receipt) and PM response (taking a photograph of it). We expected that all three encoding strategies would foster faster PM responses, compared to the standard PM instruction, since the effect of II leading to lower resource consuming, and thus presumably more automatic responding, was found for the if-then statement alone [24,40], visualisation alone [24], and the combination of the two [38].

## Method

### Design

The study was conducted as a one-way factorial design, with condition as a between-subjects factor: control condition *vs* if-then statement condition *vs* visualisation condition *vs* if-then statement with visualisation condition.

### Participants

To ensure sufficient power, we performed the a priori power analysis on GPOWER 3.1 [53]. The effect size calculation was based on the laboratory-based PM performance reported by McFarland and Glisky [24] (*f* = .366), who compared young adults across the same four conditions that were included in our design: the standard PM instruction, the if-then statement alone, visualisation alone, and the combination of the if-then statement and visualisation. With an alpha level of .05 and the minimum power of .80, 88 participants were necessary to find a statistically significant effect in the model. We recruited 121 participants to account for a possible loss of participants during the study, and some participants possibly having technical problems with taking photographs.

Participants were enrolled via the Jagiellonian University research recruitment system and via email invitations sent to students at Jesuit University Ignatianum in Krakow. Participants received credits in the recruitment system and took part in a draw for cash vouchers. Written informed consent was obtained from all participants. The informed consent stated: ‘I [*participant’s name*] voluntarily agree to participate in the research study on everyday memory that is conducted by Kaja Szarras-Kudzia. I understand that I can withdraw at any time from the study, without any consequences of any kind. I understand that all information I provide for this study will be treated confidentially. I understand that in any report on the results of this research my identity will remain anonymous, and all my responses will be treated as confidential in the manner that will prevent the identification of individual responses.’ The written ethical approval for this research was obtained from the Research Ethics Committee of the Institute of Psychology at the Jagiellonian University.

Participants were randomly assigned to one of the four conditions that differed in the PM task instruction (control condition *vs* if-then statement *vs* visualisation *vs* if-then statement with visualisation). The data of 29 participants was then excluded from the final analyses because of two reasons. First, four participants did not finish the study, either due to illness or resigning from participation. Second, 25 participants were excluded because it was not possible to calculate their response times for the PM task. This happened either because they sent photographs that were incomplete or too blurred to read the time when the receipt was being printed out, or they had a model of the mobile phone that did not store information about the time when the photographs were being made (see *The event-based PM task* section for more details). A chi-square test revealed no significant differences between the conditions in the number of participants excluded from the analyses; χ^2^(3) = 3.14, *p* = .370 (control condition– 10, if-then statement– 9, visualisation– 6, if-then statement with visualisation– 4). Likewise, the pair comparisons did not reveal any significant differences between the conditions (*p*_s_ > .120).

Finally, a total of 92 participants aged 19 to 31 (*M* = 21.83, *SD* = 2.40) were included in the analyses. Table 1 shows demographic details of the final sample. A series of one-way factorial ANOVA’s and a chi-square test (for gender) revealed no significant differences between the experimental conditions on the demographic variables.

**Table 1:** Demographic characteristics as a function of condition.

	Control	If-then statement	Visualisation	If-then statement + visualisation	Comparison
	(*n* = 23)	(*n* = 21)	(*n* = 23)	(*n* = 25)	
Age	21.70 (2.87)	22.67 (2.78)	21.91 (1.95)	21.16 (1.82)	*F*(3, 88) = 1.56, *p* = .205, η^2^_p_ = .05
Gender structure	70% female	62% female	91% female	84% female	χ^2^(3) = 6.80, *p* = .078
Education (years)	12.91 (1.20)	13.33 (2.01)	13.70 (1.84)	13.20 (2.10)	*F* < 1
Health at present	3.96 (0.64)	3.90 (0.70)	3.96 (0.71)	3.68 (0.75)	*F* < 1

*Note*. Health at present (1 *= very poor*, 5 = *very good)*.

### Materials

#### Event-based PM task

The participants were told that every time they would be shopping for the first time on a given day, they would need to remember to use their mobile phones to take a photograph of the receipt *as soon as* the receipt would be given to them. The instruction was provided to each participant on Friday, and they were asked to take a photograph each day during which they would be shopping for a duration of seven days, from the following Monday until Sunday (hereinafter referred to as ‘the study period’). It was stressed that the photograph should: (a) be taken *immediately* after receiving the receipt, (b) capture the date and time that would be printed on the receipt, (c) be taken only during the first shopping activity of the day. The experimenter also stressed that neither the bought items nor the prices on the receipt are important for the experiment, and therefore participants may cover these pieces of information, when taking the photograph, to keep them private. Participants were asked to maintain their usual shopping frequency during the study period and told that they would get an email from the experimenter after the study period asking them to send all the photographs. Participants then had a two-day delay over the weekend before the onset of the PM task. The experimenter did not contact them until after the photograph-taking period. Issuing receipts is obligatory for all sellers in Poland and all participants had smartphones with cameras, which ensured access to the crucial components of the PM task. Comparing the date and time information printed on the receipt with those included in the non-editable picture property file in the participant’ smartphone (about the exact time of taking the photograph) showed how much time elapsed between issuing the receipt and taking the photograph of it. This provided a precise and objective measure of PM performance. The pilot study revealed that participants were able to take a photograph within 20 seconds of the time the receipt was supplied.

#### Additional measures to control for the ‘busyness’ during the study period

If the participants were very busy during the study period, they would have less cognitive resources to cope with the PM task introduced by the experimenter. Therefore, we wanted to be sure that the busyness did not differ systematically across conditions. To this end, we used the Martin and Park Environmental Demands Questionnaire (Additional Measure 1) as well as we asked participants how many of their individual intentions they were planning to carry out during the study period and how important they were (Additional Measure 2). The Martin and Park Environmental Demands Questionnaire [54] provides the subjective measures for everyday busyness and routine. The Busyness scale consists of seven items (e.g., ‘How often did you have too many things to do each day to actually get them all done?’; ‘How often did you have so many things to do that you go to bed later than your regular bedtime?’) which are answered on a 5-point scale (1 = *never* to 5 = *very often*). The Routine scale consists of five items which are answered on the same scale but then reversed. We used only the Busyness scale and the instruction was modified to refer only to the study period.

Additional Measure 2 is a frequently used procedure for analysing everyday intentions (see e.g., [55–57]). Before the study period, participants were given four minutes to write as many instances as possible of the ‘jobs, appointments, and activities’ that they intended to carry out over the following week and then to rate how important the completion of each listed activity was, from 1 (*unimportant*) to 5 (*very important*). Participants used this list again, when they returned after the study period, to document completion (or noncompletion) of the activities they had intended to do during the study period. The number of intended activities, and their importance, reflected how busy the participant was within the study period. In addition, evaluating the completion of intended activities enabled us to control whether there were any systematic differences between the conditions in a general ability to handle everyday intentions that would be independent from the experimental manipulation.

### Procedure

#### First meeting: Experimental manipulation

**Fig 1:**
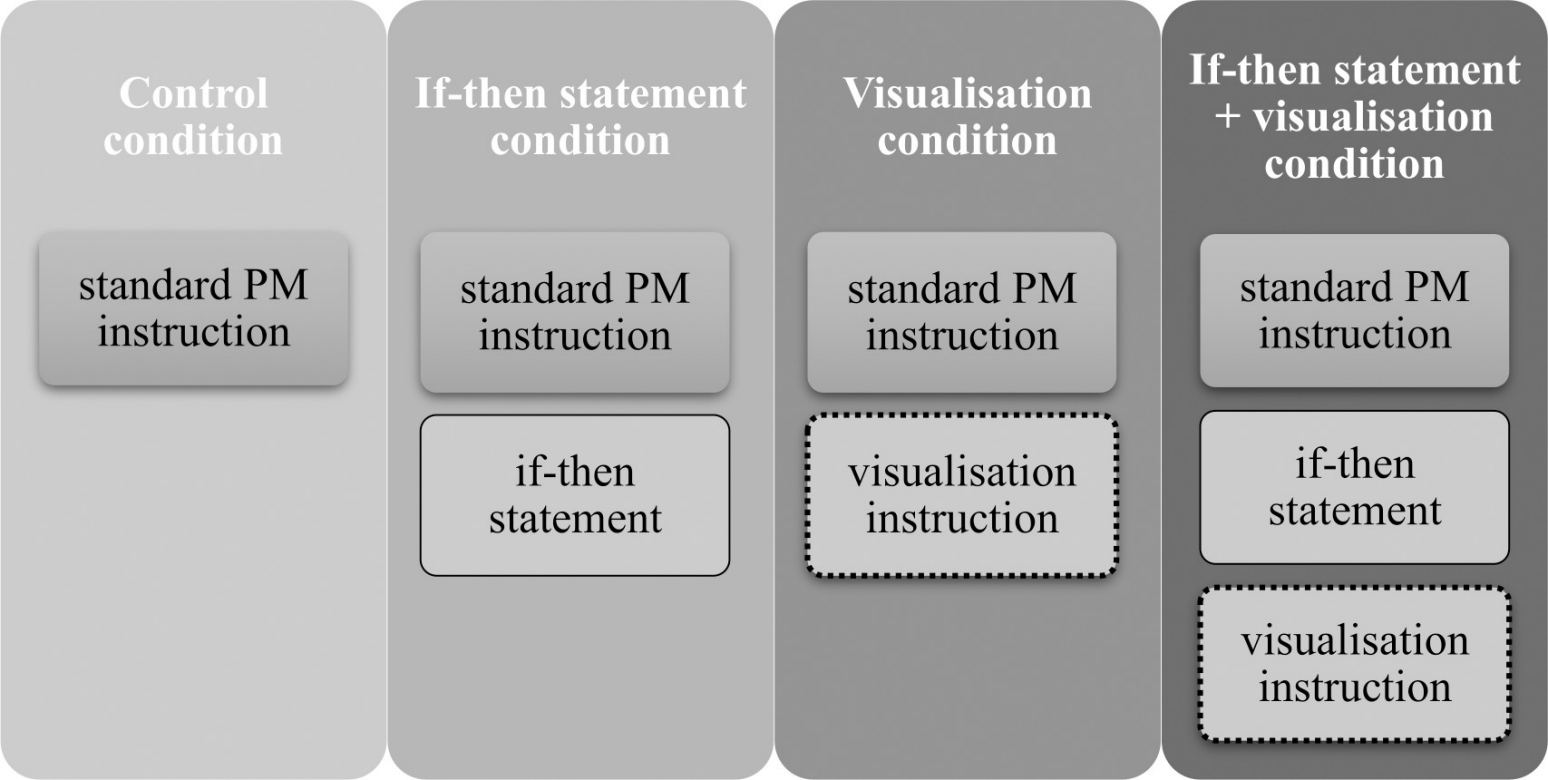
The combination of the instruction elements provided in each experimental condition.

The first meeting always took place on Friday. After arrival at the laboratory, participants filled the consent form and were shortly introduced into the purposes and outlook of the study. Next, they were given instructions and the form to write down their own intentions for the following week (i.e., for the study period) and to rate how important those intentions were (Additional Measure 2). The event-based PM task was then presented in a different manner for each experimental condition. These instruction sets were created by combining three elements: (a) the standard PM instruction, (b) the if-then statement instruction in the form of the ‘if-then’ sentence, and (c) the visualisation instruction. These elements were combined differently, depending on the experimental condition (see Fig 1 and details below). All were written on a piece of paper. *The standard PM instruction* read: ‘During next week, from Monday till Sunday, every time you will be shopping for the first time in a given day, take a picture of the receipt immediately after you receive it. Remember: once a day; first shopping activity, first receipt.’ *The if-then statement instruction* read: ‘Next week if I receive a receipt, then I will immediately take a picture of it.’ *The visualisation instruction* read: ‘Now imagine yourself at the checkout, right about to pay for your purchase. You hear other people buzzing around, you look at the person at the checkout. You talk to the cashier who gives you the total amount due and asks about the payment method. You pay and get the receipt. You can feel the thin paper in your hand. You can see black print on a white strip of paper. Now you take the mobile phone and take a picture of the receipt. Imagine it vividly: hear sounds, feel smells, touch objects. Imagine that now for at least 30 seconds.’

In the *control condition*, participants were presented with the standard PM instruction in a written form, and then asked to repeat it until they were able to write down the instruction in their own words, without consulting the instruction provided by the experimenter. In the *visualisation condition*, participants were presented with the standard PM instruction, and asked to repeat it until they were able to write it down without help, exactly as in the *control condition*. Next, they were given the visualisation instruction and asked to engage in the visualisation process for at least 30 seconds. In the *if-then statement condition*, participants were presented with the standard PM instruction in a written form. Next, the written ‘if-then’ statement was given to them, and they were asked to repeat it until they were able to write down the statement without any help. In the *if-then statement plus visualisation condition*, participants were encoding the PM task in the same way as in the if-then statement condition, which was followed by the 30-second visualisation conducted in the same way as in the visualisation condition. The experimental manipulation was restricted only to the phase of encoding the PM task.

After the PM task encoding phase, all participants were given an opportunity to ask questions about the instructions. At the end, the experimenter emphasized the most important aspects of the PM task, as described in the event-based PM task section. Other issues, such as the participants’ shopping habits and the need for their mobile phone pictures to be able to record the date and time, were not mentioned at this point to avoid influencing participants’ spontaneous behaviours during the study period.

#### The study period

Participants needed to remember to start the event-based PM task on the following Monday, after a two-day delay over the weekend, and to continue to take photographs of the first receipt received on each day when they did shopping, until Sunday. The experimenter neither reminded them to start the PM task nor contacted them during the study period.

#### An email request to send photographs and fill in the online form

On Monday following the study period, participants received an email from the experimenter requesting them to send her all the photographs of the receipts that had been taken during the study period, and to answer the questions embedded into the online form. The form included questions about demographic details and the Busyness scale (Additional Measure 2). Finally, the date for the second meeting was arranged.

#### Second meeting

**Fig 2:**
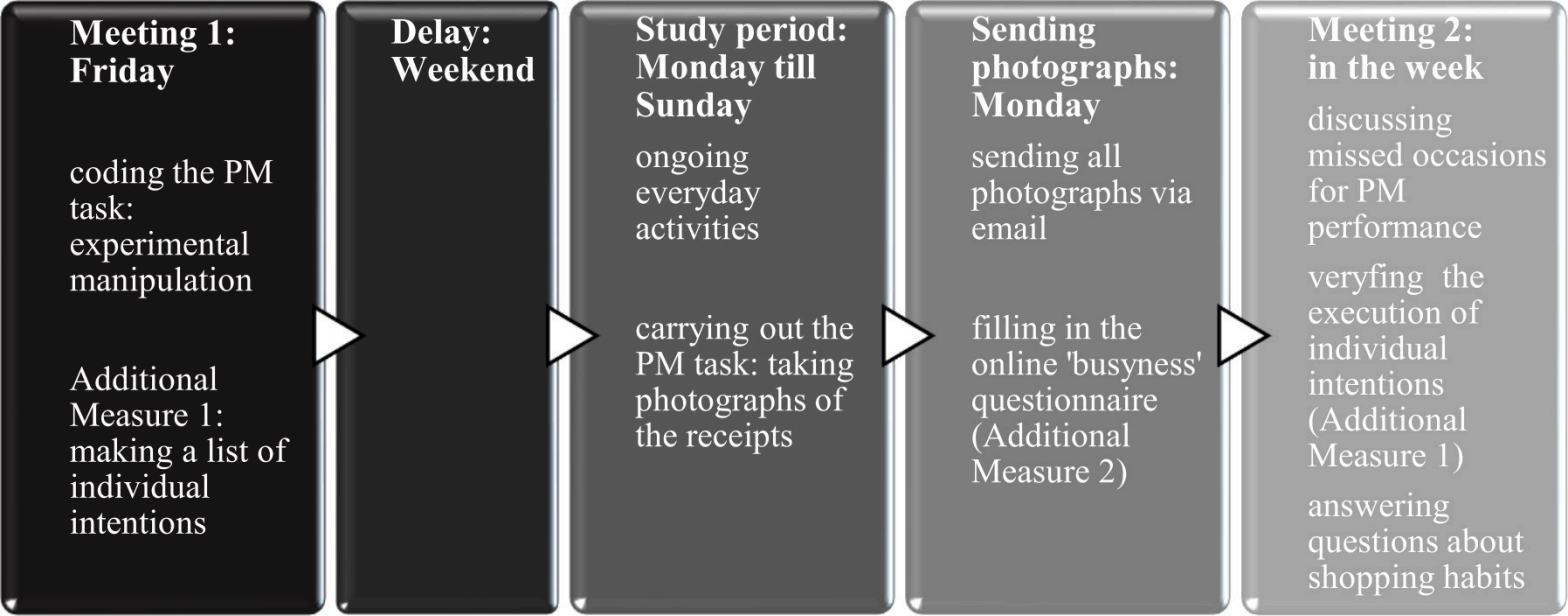
A summary of the experimental procedure.

The meeting took place 10 to 14 days after the first meeting, and no longer than five days after the end of the study period. First, participants were given the list of their individual intentions for the study period that they had made during the first meeting and asked which of them they had actually carried out (Additional Measure 1). Second, they were asked to write, in their own words, the content of the task they had been given by the experimenter at the first meeting. This open question served to control whether a failure to perform the PM task by a given participant might have been due to a retrospective memory error, i.e., the participant entirely forgot that they were supposed to take photographs, rather than a prospective memory error, i.e., the participant forgot to take photographs at the moments when they were being given the receipt (see [25,43,58] for the same procedure). All participants remembered the content of the PM task. Third, the experimenter took the participants through a table in which all days of the study period were included, together with the information of whether participants had sent a photograph on a given day. If the photograph from a given day had not been sent, they were asked whether they had been shopping on that day, and if yes, why they had not provided a picture. Finally, a few questions regarding participants’ shopping habits were asked. Some shopping habits might have made the PM task easier for participants, and we wanted to be sure that experimental conditions did not differ systematically in this respect. For instance, the task might have been easier for the participants who customarily take receipts, as compared to those who would rather leave them with a cashier, as the latter participants might have needed to additionally remember to take a receipt. Furthermore, it might be that the participants who customarily pay with a card rather than cash pay more attention to receipts. It might also be that some participants intentionally increased the frequency of their shopping during the study period to provide themselves with the opportunities to make photographs of the receipts. Therefore, participants were asked to indicate: (a) whether their shopping frequency during the study period changed in comparison to their usual shopping frequency (decreased, remained the same or increased); (b) what their usual payment method is (credit card or cash), and (c) whether they usually take a receipt or leave it with a cashier. When all questions were answered, participants were thanked and debriefed. A summary of the experimental procedure is presented on Fig 2.

## Results

All statistical analyses were conducted using the *Statistica 13*.*3*. The effect size was measured by partial eta squared (η^2^_p_) with small, medium, and large effects defined as .01, .06, and .16, respectively [59]. As recommended for multiple planned comparisons [60], the Bonferroni correction for multiple comparisons was used, i.e., alphas were divided by the number of planned comparisons tested for a given factor.

### Homogeneity of the conditions in terms of the shopping habits, and shopping during the study period

Table 2 shows details of the shopping habits and mean scores on the measures of busyness and handling everyday intentions as a function of condition. With regard to how participants usually do shopping, a substantial majority of them in each condition customarily take receipts rather than leave them with the cashier, and pay with a credit card rather than cash. For the study period, a huge majority of participants in each condition shopped with the same or lower frequency than usual, as compared to those who increased the shopping frequency during the study period. Importantly, the cross-tabulation tests did not reveal any significant differences between conditions in the shopping habits and the frequency of shopping during the study period as compared to a usual frequency (*p*_*s*_ > .300). In addition, we compared the conditions in terms of the number of shopping days during the study period, and whether participants started shopping on Monday. Shopping rates were taken into account because each successful PM task may strengthen associations for subsequent tasks. Thus, participants that went shopping less frequently might have had less opportunities for strengthening these associations than did participants who went shopping more frequently. Furthermore, the delay between the instruction (i.e., experimental manipulation) and task onset depended on when participants went shopping for the first time. The conditions did not differ either in shopping rates or the proportions of participants who went shopping on Monday (*p*_*s*_ > .532).

**Table 2:** Shopping habits and busyness measures as a function of condition.

	Control	If-then statement	Visualisation	If-then statement + visualisation	Comparison
	(*n* = 23)	(*n* = 21)	(*n* = 23)	(*n* = 25)	
Taking receipts (%)	65	67	57	80	χ^2^(3) = 3.10, *p* = .377
Paying with a card (%)	87	90	91	84	χ^2^(3) = 0.77, *p* = .858
The same or lower shopping rate (%)	74	95	83	80	χ^2^(3) = 3.66, *p* = .301
Number of shopping days in the study period	5.26 (1.25)	4.71 (1.27)	5.17 (1.23)	4.96 (1.31)	*F* < 1
Shopping started on Monday (%)	70	62	78	60	χ^2^(3) = 2.20, *p* = .533
MPED Busyness	23.04 (5.46)	24.57 (5.55)	25.52 (3.64)	24.84 (4.71)	*F*(3, 88) = 1.06, *p* = .370, η^2^_p_ = .04
Number of intentions	9.74 (3.83)	8.62 (2.87)	8.65 (2.55)	8.20 (2.29)	*F*(3, 88) = 1.18, *p* = .322, η^2^_p_ = .04
Importance of intentions	3.75 (0.43)	3.91 (0.34)	3.92 (0.46)	3.96 (0.57)	*F* < 1
Proportion of executed intentions	0.72 (0.18)	0.71 (0.17)	0.73 (0.14)	0.67 (0.16)	*F* < 1

*Note*. MPED Busyness = the Busyness scale of the Martin and Park Environmental Demands Questionnaire (1 = *never*, 5 = *very often*); Importance of intentions (1 *= unimportant*, 5 = *very important)*; The executed intentions = the proportion of the executed individual activities out of all planned by the participant for the study period.

Participants had 463 shopping occasions in total, i.e., they went shopping on the day which was indicated either by them sending a photograph of the receipt from that day or, even if they did not send the photograph at all, they acknowledged that they had gone shopping on that day. For 70 (15%) shopping occasions, the photograph of the receipt was not sent to the experimenter. For all these cases, participants indicated memory failure as a reason for not sending the photograph, i.e., they entirely forgot to take a picture (39% out of 70), they forgot to take a picture when shopping and could not find the receipt later during the day (21% out of 70), or they forgot to take the receipt from the cashier (40% out of 70). None of the participants mentioned that it was difficult for them to take a picture due to, for example, being distracted when paying or just after that (distracted by other people, phone calls, incoming messages etc.) or due to technical problems with their mobile. Therefore, all days on which participants went shopping, even if they did not send the photograph at all, were taken into account when calculating their PM performance. The conditions did not differ in the proportions of the three reasons provided for not sending the photographs, p_s_ > .094.

The conditions did not differ in the level of busyness during the study period, either as measured with the Busyness scale of the Martin and Park Environmental Demands questionnaire (*p* = .370), or as measured by the number (*p* = .322) and importance of activities that participants had been planned for the study period (*F* < 1). The level of execution of participants’ everyday intentions was also the same across conditions (*F* < 1). In conclusion, potential differences between the conditions in how well participants performed the PM task of taking photographs of the receipts could not be explained by differences in the shopping habits, shopping patterns during the study period, differences in a general ability of handling everyday intentions, or how busy participants were during the study period (either in general or with other intended activities).

### PM Performance

To set a time frame for correct PM responses, we took advantage of the criterion applied in previous naturalistic PM studies, including those on the II effectiveness, in which a 10-minute window had been typically used, across various types of naturalistic PM tasks [e.g., 22,26,27,61–64]. By choosing a 10-minute window as a time frame, we were thus following common practice in PM studies in which PM tasks are assigned by the experimenter to be completed outside the lab. In our study, the PM response was considered correct (the ‘hit’) if it occurred within 10 minutes after finalising the shopping, i.e., the difference between the time and date stamp of the photograph file and the time printed on the receipt was not longer than 10 minutes.

Two measures of PM performance were employed: the proportions of correct PM responses and the reaction times for correct PM responses. The proportions of correct PM responses showed how often participants remembered to perform the intended action within the set time frame, whereas reaction times showed how immediate these responses were. Reaction times were used to investigate whether the encoding techniques would lead to more automatic PM responses, which would be particularly beneficial to PM tasks with a narrow time window for making an appropriate response. The basis for both measures was the calculation of time that had elapsed before a picture of the receipt was taken. For each PM response (i.e., the picture of the receipt) the time was calculated in seconds as the difference between the time of taking a photograph (the non-editable data in the picture’s meta-properties information) and the time when the receipt was issued (the time and date print visible in the receipt itself). For example, when the time and date printed on the photographed receipt was ‘December 21^st^; 11:10:00’, and the time and date of taking the picture, that was marked in the picture property file, was ‘December 21^st^, 11:15:49’, the time elapsed was 349 seconds.

Sixteen participants (17% of 92 participants) decided not to show the bought items and/or prices on some of their receipts.

#### The proportions of correct PM responses

The proportion of correct PM responses was calculated by dividing the number of hits by the number of the PM occasions, i.e., the number of times the participant did shop during the study period. The mean proportions of correct PM responses were entered into a one-way ANOVA which revealed no differences between the conditions, *F* < 1 (see Table 3). To investigate whether the results may change with a more lenient time frame for correct responding, we increased the size of the correct time window to 15 minutes (see Liu and Park [26] for the same strategy of analysing the data in the naturalistic experiment). The results were unchanged, *F*(3, 88) = 1.44, *p* = .237, η^2^_p_ = .05, indicating no differences between the conditions.

**Table 3:** PM performance as a function of condition.

	Control	If-then statement	Visualisation	If-then statement + visualisation
	(*n* = 23)	(*n* = 21)	(*n* = 23)	(*n* = 25)
Proportion of correct PM responses	0.38 (0.31)	0.34 (0.39)	0.45 (0.26)	0.40 (0.28)
Reaction times for correct PM responses (*sec*.)	238.81 (154.61)	107.25 (80.54)	160.08 (94.09)	159.69 (133.70)

*Note*. The PM response was considered correct if it occurred within 10 minutes after the receipt was issued.

Out of all control variables measured (see Table 2), three were significantly related to PM performance as measured by the proportions of correct PM responses. These proportions were higher among participants who customarily take receipts, *F*(1, 90) = 4.04, *p* = .047, η^2^_p_ = .04, who went shopping on Monday *F*(1, 90) = 10.86, *p* = .001, η^2^_p_ = .11, as well as with higher shopping rates in the study period, *r* = .23, *p* = .030. However, including these variables as either another between-subjects factor (whether customarily take receipts, whether went shopping on Monday) or a covariate (shopping rates in the study period) in an ANOVA with condition as the between-subjects factor did not change the results, i.e., the conditions did not differ in the mean proportions of correct PM responses (*p*_*s*_ > .256). The control variables did not interact with condition either, (*p*_*s*_ > .083).

#### Reaction times for correct PM responses

The mean reaction times, measured in seconds, for correct PM responses (those made within the 10-minutes time frame) were entered in a one-way ANOVA which revealed the main effect of condition, *F*(3, 65) = 3.05, *p* = .035, η^2^_p_ = .12 (see Table 3). Planned comparisons, with adjusted alpha level of .017 (.05/3), revealed that, as predicted, reaction times were significantly faster in the if-then statement condition than in the control condition *F*(1, 65) = 8.37, *p* = .005, η^2^_p_ = .11. Reaction times were not faster in the two remaining encoding strategy conditions, as compared to the control condition: the if-then statement with visualisation, *F*(1, 65) = 3.89, *p* = .053, η^2^_p_ = .06; visualisation, *F*(1, 65) = 3.95, *p* = .051, η^2^_p_ = .06.

Again, to test the robustness of this finding, we increased the size of the correct time window to 15 minutes. The similar pattern of results was obtained, with the main effect of condition, *F*(3, 70) = 3.18, *p* = .029, η^2^_p_ = .12, and reaction times faster in the if-then condition compared to the control condition, *F*(1, 70) = 7.87, *p* = .007, η^2^_p_ = .10. Reaction times were not faster in the two remaining encoding strategy conditions, as compared to the control condition: the if-then statement with visualisation, *F*(1, 70) = 2.94, *p* = .091, η^2^_p_ = .04; visualisation, *F* < 1.

None of the control variables measured (see Table 2) was related to PM performance as measured by the reaction times for correct PM responses (*p*_*s*_ > .168).

## Discussion

The aim of this present study was to investigate the benefits of using the if-then statement alone, visualisation alone, and the combination of the two for the PM performance of young adults in everyday life. The if-then statement presented alone speeded up correct PM responses to the target event, compared to the standard PM instruction, across both more strict and more lenient time frames for correct responding. These results are in line with the theoretical proposal put forward by several authors [9,38,40] that II stimulates a strong associative encoding between an anticipated situation and an intended action, thereby promoting more reflexive and automatic triggering of the intended action when the critical situation is encountered. Our findings complement the results of the studies which showed this increase in automaticity as measured by the amount of cognitive resources required [24,38,40], by showing the same effect with a different measure of automaticity, i.e., response speed.

Contrary to expectations, the if-then statement presented alone was the only encoding strategy that significantly speeded up correct PM responses to the target event, as compared to the standard PM instruction. The impact of visualisation in fostering immediate responses might not have been as strong as the impact of the if-then statement, as the positive effects of visualisation may depend on how accurately and in detail the person is able to visualise situations in which they will need to remember to do something (see [23] for a similar argument). It is much easier to precisely visualise those situations in laboratory settings as, in contrast to everyday settings, nothing much changes in the surroundings between encoding the PM task and the opportunity to carry it out. Our participants might have tried to imagine the shop that they most often go to and the cashier whom they most often see there, but the circumstances of their shopping activities probably change considerably from one day to another, with different shops, different cashiers and different people around.

Surprisingly, the impact of the if-then statement on response speed was evident when the statement was presented alone, but not when it was followed by the visualisation of carrying out the PM task. However, it is worth noting that in most studies showing the effectiveness of the if-then statement and visualisation combined for PM improvement among young adults, the if-then statement was presented after visualisation or simultaneously with visualisation ([38] Experiment 1 & 2, [24,30,33,65]). In contrast, we asked the participants to imagine how they are carrying out the PM task after the if-then statement had been presented. Since visualisation was long and very detailed, and the if-then statement was short and written down by the participants just once, they might have remembered just visualisation, i.e., imagery might have interfered with the statement or overwritten it. If this was the case, the combined condition would work as the condition with visualisation alone.

It is worth noting that the pattern of results, i.e., PM response being faster after the if-then statement alone, compared to the control condition, with no effects of visualisation alone and the combination of visualisation and the if-then statement, speaks against a possible interpretation that faster responses can be explained by the group differences in the instruction elaboration. The control condition and the if-then statement condition hardly differed in terms of the instruction elaboration. In both conditions participants were repeating the instruction (the standard instruction in the control condition and the if-then statement in the if-then statement condition) until they were able to write it down correctly without any help. In contrast, an increase in the instruction elaboration, compared to the control condition, was substantial for the visualisation condition and the if-then statement with visualisation condition. Still the latter two conditions did not produce faster PM responses, as compared to the control condition.

Contrary to predictions, we have not found any impact of the encoding strategies on how often the participants remembered to take a photograph of the receipt. The instruction conditions did not have any effect on the proportions of accurate responses within 10 or 15 minutes of the target event even though, based on the size of the effect reported by McFarland and Glisky [24] (*f* = .366), our final sample (N = 92) was sufficient to detect the effect if it was present, with an alpha level of .05 and the minimum power of .80. Furthermore, the performance on our experimental task left room for improvement, since the proportion of accurate responses under the standard PM instruction was just 38%. However, it is worth noting that the power analysis was based on the laboratory-based study, and our investigation was the first attempt to replicate the effect for the PM task performed by young adults in naturalistic settings. It may be that the effect is more difficult to detect in more complex and less controllable circumstances of everyday life. Accordingly, we found that the proportions of accurate responses were influenced by some aspects of the participants’ shopping habits and their shopping rate in the study period. Such differences in natural environment and behaviour are difficult to eliminate in naturalistic experiments, and they might have attenuated the impact of our manipulation. In contrast, we were able to demonstrate the impact of manipulation on response speed, which measure was not dependent on differences in shopping habits or shopping frequency.

Furthermore, for the intended action to be performed, an individual needs to notice a prespecified cue first, and then to recognise the cue as the PM target event, i.e., needs to remember that a certain action should be carried out. The pattern of results, i.e., no effect of the if-then statement, as an isolated strategy, on the proportion of correct responses but a significant effect of this strategy on response speed, suggests that the statement did not help in the first stage of PM performance, but was helpful in the second stage. In other words, it does not seem that the if-then statement alone led to increased cue saliency, but once the participants paid attention to the cue, those provided with that strategy more quickly remembered about the required action, probably due to a stronger association between the cue and the intention. It is worth noting that participants may be more occupied with ongoing tasks in the naturalistic settings, compared to the laboratory conditions, when the critical cue appears. In our study, when receipts were being issued, the participants were probably very busy with packing the shopping items, hiding the wallet and making room for a next client at the cashier. Furthermore, the prespecified cue, i.e., the receipt, was neither a distinct nor important element of the situation itself, without the PM component of it. Future studies may address the issue whether the if-then statement may help young adults to more often remember about the intended action in naturalistic context if more salient cues are used.

Nevertheless, our findings suggest that the if-then statement, presented alone, may be effective in speeding up PM responses. As many everyday PM tasks have a very narrow window of opportunity, i.e., for the task to be successfully accomplished the person needs to respond immediately when the specified opportunity appears, our results suggest that that strategy may help in many everyday circumstances. These will not be just those everyday duties that inspired us when developing the experimental task, e.g., validating a free car park ticket just before a one-way exit from a sport facility or a supermarket. Even for the intentions, such as posting a letter when seeing the post office, or passing on a message to a colleague when seeing her, a prompt response is essential. Retrieving the intention even a few minutes after seeing the PM target means that the person has already gone past the post office or that the colleague has already walked away, and that the opportunity has been missed.

## References

[1] Einstein GO, McDaniel MA. Prospective memory: Multiple retrieval processes. Curr Dir Psychol Sci. 2005;14:286–290. doi: 10.1111/j.0963-7214.2005.00382.x

[2] Haas M, Zuber S, Kliegel M, Ballhausen N. Prospective memory errors in everyday life: Does instruction matter? Memory. 2020;28:196–203. doi: 10.1080/09658211.2019.1707227 31893967

[3] Niedźwieńska A, Sołga J, Zagaja P, Żołnierz M. Everyday memory failures across adulthood: Implications for the age prospective memory paradox. PLoS ONE. 2020;15(9):e0239581. doi: 10.1371/journal.pone.0239581 32976533 PMC7518607

[4] Dismukes RK. Prospective memory in workplace and everyday situations. Curr Dir Psychol Sci. 2012;21(4):215–220. doi: 10.1177/0963721412447621

[5] Stone M, Dismukes K, Remington R. Prospective memory in dynamic environments: Effects of load, delay, and phonological rehearsal. Memory. 2001;9(3):165–176. doi: 10.1080/09658210143000100 11469311

[6] McDaniel MA, Einstein GO. Prospective memory: An overview and synthesis of an emerging field. Thousand Oaks, CA: Sage Publications; 2007.

[7] Anderson FT, Strube M J, McDaniel MA. Toward a better understanding of costs in prospective memory: A meta-analytic review. Psychol Bull. 2019;145(11):1053–1081. doi: 10.1037/bul0000208 31464456

[8] Gollwitzer PM. Goal achievement: The role of intentions. Eur Rev Soc Psychol. 1993;4(1):141–185. doi: 10.1080/14792779343000059

[9] Gollwitzer PM. Implementation intentions: Strong Effects of Simple Plans. Am Psychol. 1999;54(7):493–503. doi: 10.1037/0003-066X.54.7.493

[10] Gollwitzer PM, Sheeran P. Implementation intentions and goal achievement: A meta‐analysis of effects and processes. Adv Exp Soc Psychol. 2006;38:69–119. doi: 10.1016/S0065-2601(06)38002-1

[11] Gollwitzer PM, Brandstätter V. Implementation intentions and effective goal pursuit. J Pers Soc Psychol. 1997;73(1):186–199. doi: 10.1037/0022-3514.73.1.186

[12] Achtziger A, Gollwitzer PM, Sheeran P. Implementation intentions and shielding goal striving from unwanted thoughts and feelings. Pers Soc Psychol Bull. 2008;34(3):381–393. doi: 10.1177/0146167207311201 18272806

[13] Norman P, Webb TL, Millings A. Using the theory of planned behaviour and implementation intentions to reduce binge drinking in new university students. Psychol Health. 2019;34(4):478–496. doi: 10.1080/08870446.2018.1544369 30636436

[14] Chapman J, Armitage CJ, Norman P. Comparing implementation intention interventions in relation to young adults’ intake of fruit and vegetables. Psychol Health. 2009;24(3):317–332. doi: 10.1080/08870440701864538 20204996

[15] Milne S, Orbell S, Sheeran P. Combining motivational and volitional interventions to promote exercise participation: Protection motivation theory and implementation intentions. Br J Health Psychol. 2002;7(2):163–184. doi: 10.1348/135910702169420 14596707

[16] Kretschmer A, Altgassen M, Rendell PG, Bölte S. Prospective memory in adults with high-functioning autism spectrum disorders: Exploring effects of implementation intentions and retrospective memory load. Res Dev Disabil. 2014;35(11):3108–3118. doi: 10.1016/j.ridd.2014.07.052 25151603

[17] Khoyratty NB, Wang Y, O’Gorman JG, Lloyd C, Williams PL, Chan RC, et al. Forming implementation intentions improves prospective memory in early psychosis. Psychiatry Res. 2015;228(3):265–271. doi: 10.1016/j.psychres.2015.05.101 26142837

[18] Lee JH, Shelton JT, Scullin MK, McDaniel MA. An implementation intention strategy can improve prospective memory in older adults with very mild Alzheimer’s disease. Br J Clin Psychol. 2016;55(2):154–166. doi: 10.1111/bjc.12084 25994043 PMC4654698

[19] Foster ER, McDaniel MA, Rendell PG. Improving prospective memory in persons with Parkinson disease: A randomized controlled trial. Neurorehabil Neural Repair. 2017;31(5):451–461. doi: 10.1177/1545968317690832 28176547 PMC5393947

[20] Liu LL, Wang Y, Cui JF, Li Y, Yang TX, Chen T, et al. The effect of implementation intentions on prospective memory performance in patients with schizophrenia: A multinomial modeling approach. Schizophr Res. 2020;215:120–125. doi: 10.1016/j.schres.2019.11.003 31784339

[21] Schnitzspahn KM, Kliegel M. Age effects in prospective memory performance within older adults: The paradoxical impact of implementation intentions. Eur J Ageing. 2009;6(2):147–155. doi: 10.1007/s10433-009-0116-x 28798601 PMC5547303

[22] Brom SS, Kliegel M. Improving everyday prospective memory performance in older adults: comparing cognitive process and strategy training. Psychol Aging. 2014;29(3):744–755. doi: 10.1037/a0037181 25244491

[23] Burkard C, Rochat L, Blum A, Emmenegger J, Juillerat Van der Linden AC, Van der Linden M. A daily-life-oriented intervention to improve prospective memory and goal-directed behaviour in ageing: A pilot study. Neuropsychol Rehabil. 2014;24(2):266–295. doi: 10.1080/09602011.2014.887023 24559524

[24] McFarland C, Glisky E. Implementation intentions and imagery: Individual and combined effects on prospective memory among young adults. Mem Cognit. 2012;40(1):62–69. doi: 10.3758/s13421-011-0126-8 21732204

[25] Zimmermann TD, Meier B. The effect of implementation intentions on prospective memory performance across the lifespan. Appl Cogn Psychol. 2010;24(5):645–658. doi: 10.1002/acp.1576

[26] Liu LL, Park DC. Aging and medical adherence: the use of automatic processes to achieve effortful things. Psychol Aging. 2004;19(2):318–325. doi: 10.1037/0882-7974.19.2.318 15222825

[27] Brom SS, Schnitzspahn KM, Melzer M, Hagner F, Bernhard A, Kliegel M. Fluid mechanics moderate the effect of implementation intentions on a health prospective memory task in older adults. Eur J Ageing. 2013;11(1):89–98. doi: 10.1007/s10433-013-0288-2 28804317 PMC5549189

[28] Niedźwieńska A, Barzykowski K. The age prospective memory paradox within the same sample in time-based and event-based tasks. Aging Neuropsychol Cogn. 2012;19(1–2):58–83. doi: 10.1080/13825585.2011.628374 22112250

[29] Rendell PG, Thomson DM. Aging and prospective memory: Differences between naturalistic and laboratory tasks. J Gerontol *B* Psychol Sci Soc Sci. 1999;54(4):P256–P269. doi: 10.1093/geronb/54b.4.p256 12382595

[30] Meeks JT, Marsh RL. Implementation intentions about nonfocal event-based prospective memory tasks. Prof Psychol Res Pr. 2010;74(1):82–89. doi: 10.1007/s00426-008-0223-x 19130080

[31] Brewer GA, Ball BH, Knight JB, Dewitt MR, Marsh RL. Divided attention interferes with fulfilling activity-based intentions. Acta Psychol. 2011;138(1):100–105. doi: 10.1016/j.actpsy.2011.05.011 21704959

[32] Rummel J, Einstein GO, Rampey H. Implementation-intention encoding in a prospective memory task enhances spontaneous retrieval of intentions. Memory. 2012;20(8):803–817. doi: 10.1080/09658211.2012.707214 22897132

[33] Smith RE, Rogers MDM, McVay JC, Lopez JA, Loft S. Investigating how implementation intentions improve non-focal prospective memory tasks. Conscious Cogn. 2014;27:213–230. doi: 10.1016/j.concog.2014.05.003 24929276 PMC4113409

[34] McCrea SM, Penningroth SL, Radakovich MP. Implementation intentions forge a strong cue–response link and boost prospective memory performance. J Cogn Psychol. 2015;27(1):12–26. doi: 10.1080/20445911.2014.975816

[35] McDaniel MA, Scullin MK. Implementation intention encoding does not automatize prospective memory responding. Mem Cognit. 2010;38(2):221–232. doi: 10.3758/MC.38.2.221 20173194

[36] Bennett LR, Clawson DM, Kardiasmenos KS. The retrospective component of prospective memory: Relationship to prospective component success. 2005, Nov. Poster presented at the 46th Annual Meeting of the Psychonomic Society, Toronto.

[37] Kardiasmenos KS, Clawson DM, Wilken JA, Wallin MT. Effects of implementation intentions on prospective memory performance. 2004, Nov. Poster presented at the 45th Annual Meeting of the Psychonomic Society, Minneapolis, MN.

[38] McDaniel MA, Howard DC, Butler KM. Implementation intentions facilitate prospective memory under high attention demands. Mem Cognit. 2008;36(4):716–724. doi: 10.3758/mc.36.4.716 18604955

[39] Breneiser JE. Implementation Intentions and Generative Strategies in Prospective Memory Retrieval. N Am J Psychol. 2009;11(2):401–418.

[40] Cohen A-L, Gollwitzer PM. The cost of remembering to remember: Cognitive load and implementation intentions influence ongoing task performance. In: Kliegel M, McDaniel MA, Einstein GO, editors. Prospective memory: Cognitive, neuroscience, developmental, and applied perspectives. New York: Taylor & Francis Group/Lawrence Erlbaum Associates; 2008. pp. 367–390.

[41] Weinberger J, Stoycheva V. The unconscious: Theory, research, and clinical implications. Guilford Publication; 2019.

[42] Maylor EA, Smith G, Della Sala S, Logie RH. Prospective and retrospective memory in normal aging and dementia: An experimental study. Mem Cognit. 2002;30(6):871–884. doi: 10.3758/bf03195773 12450091

[43] Kliegel M, Jäger T. Delayed–execute prospective memory performance: The effects of age and working memory. Dev Neuropsychol. 2006;30(3):819–843. doi: 10.1207/s15326942dn3003_4 17083295

[44] Kelly AJ, Hertzog C, Hayes MG, Smith AD. The effects of age and focality on delay-execute prospective memory. Aging Neuropsychol Cognit. 2013;20(1):101–124. doi: 10.1080/13825585.2012.691152 22639931 PMC3432142

[45] Strickland L, Elliott D, Wilson MD, Loft S, Neal A, Heathcote A. Prospective memory in the red zone: Cognitive control and capacity sharing in a complex, multi-stimulus task. J Exp Psychol Appl. 2019;25(4):695–715. doi: 10.1037/xap0000224 30985156

[46] O’Connor AM, Campbell KL, Mahy CE. Younger and older adults’ prospective memory: the role of delay task difficulty. Aging Neuropsychol Cognit. 2021;28(2):184–200. doi: 10.1080/13825585.2020.1724866 32022629

[47] Aarts H, Dijksterhuis AP, Midden C. To plan or not to plan? Goal achievement or interrupting the performance of mundane behaviors. Eur J Soc Psychol. 1999;29(8):971–979. doi: 10.1002/(SICI)1099-0992(199912)29:8<971::AID-EJSP963>3.0.CO;2-A 25855820

[48] Achtziger A, Bayer UC, Gollwitzer PM. Committing to implementation intentions: Attention and memory effects for selected situational cues. Motiv Emot. 2012;36(3):287–300. doi: 10.1007/s11031-011-9261-6

[49] Webb TL, Sheeran P. Mechanisms of implementation intention effects: The role of goal intentions, self‐efficacy, and accessibility of plan components. Br J Soc Psychol. 2008;47(3):373–395. doi: 10.1348/014466607X267010 18096108

[50] Schneider W, Shiffrin RM. Controlled and automatic human information processing: 1. Detection, search, and attention. Psychol Rev. 1977;84:1–66. doi: 10.1037/0033-295X.84.1.1

[51] Shiffrin RM, Dumais S, Schneider W. Characteristics of automatism. In: Long L, Baddeley A, editors. Attention and performance IX. Hillsdale, N.J: Erlbaum, 1981.

[52] Meier B, Morger V, Graf P. Competition between automatic and controlled processes. Conscious Cogn. 2003;12(2):309–319. doi: 10.1016/s1053-8100(02)00069-7 12763011

[53] Faul F, Erdfelder E, Lang A-G, Buchner A. G*Power 3: A flexible statistical power analysis program for the social, behavioral, and biomedical sciences. Behav Res Methods. 2007;39:175–191. doi: 10.3758/bf03193146 17695343

[54] Martin M, Park DC. The Martin and Park Environmental Demands (MPED) Questionnaire: Psychometric properties of a brief instrument to measure self-reported environmental demands. Aging Clin Exp Res. 2003;15(1):77–82. doi: 10.1007/BF03324483 12841422

[55] Freeman JE, Ellis JA. The intention‐superiority effect for naturally occurring activities: The role of intention accessibility in everyday prospective remembering in young and older adults. Int J Psychol. 2003;38(4):215–228. doi: 10.1080/00207590344000141

[56] Marsh RL, Hicks JL, Landau JD. An investigation of everyday prospective memory. Mem Cognit. 1998;26(4):633–643. doi: 10.3758/bf03211383 9701955

[57] Niedźwieńska A, Janik B, Jarczyńska A. Age-related differences in everyday prospective memory tasks: The role of planning and personal importance. Int J Psychol. 2013;48(6):1291–1302. doi: 10.1080/00207594.2012.752097 23305040

[58] Jäger T, Kliegel M. Time-based and event-based prospective memory across adulthood: Underlying mechanisms and differential costs on the ongoing task. J Gen Psychol. 2008;135(1):4–22. doi: 10.3200/GENP.135.1.4-22 18318405

[59] Cohen J. Statistical power analysis for the behavioural sciences. 2nd ed. Hillsdale, NJ: Lawrence Erlbaum Associates; 1988.

[60] Pedhazur EJ, Schmelkin LP. Measurement, design, and analysis: An integrated approach. Hillsdale NJ: Lawrence Erlbaum Associates; 1991.

[61] Kvavilashvili L, Fisher L. Is time-based prospective remembering mediated by self-initiated rehearsals? Role of incidental cues, ongoing activity, age, and motivation. J Exp Psychol Gen. 2007;136(1):112–132. doi: 10.1037/0096-3445.136.1.112 17324087

[62] Jeong JM, Cranney J. Motivation, depression, and naturalistic time-based prospective remembering. Memory. 2009;17(7):732–741. doi: 10.1080/09658210903074673 19637094

[63] Cavuoto MG, Ong B, Pike KE, Nicholas CL, Kinsella GJ. Naturalistic prospective memory in older adults: Predictors of performance on a habitual task. Neuropsychol Rehabil. 2017;27(5):744–758. doi: 10.1080/09602011.2015.1074590 28480820

[64] Schnitzspahn KM, Kvavilashvili L, Altgassen M. Redefining the pattern of age-prospective memory-paradox: new insights on age effects in lab-based, naturalistic, and self-assigned tasks. Psychol. Res. 2020;84:1370–1386. doi: 10.1007/s00426-018-1140-2 30588544 PMC7271051

[65] Brewer GA, Marsh RL. On the role of episodic future simulation in encoding of prospective memories. Cogn Neurosci. 2010;1(2):81–88. doi: 10.1080/17588920903373960 24168273

